# Angling for Uniqueness in Enzymatic Preparation of Glycosides

**DOI:** 10.3390/biom3020334

**Published:** 2013-06-13

**Authors:** Antonio Trincone

**Affiliations:** Institute of Biomolecular Chemistry, National Research Council, Via Campi Flegrei, 34, Pozzuoli 80078, Naples, Italy; E-Mail: antonio.trincone@icb.cnr.it; Tel./Fax: +39-081-867-5095

**Keywords:** biocatalysis, glycosides, enzymes, glycosidases, glycosynthases

## Abstract

In the early days of biocatalysis, limitations of an enzyme modeled the enzymatic applications; nowadays the enzyme can be engineered to be suitable for the process requirements. This is a general bird’s-eye view and as such cannot be specific for articulated situations found in different classes of enzymes or for selected enzymatic processes. As far as the enzymatic preparation of glycosides is concerned, recent scientific literature is awash with examples of uniqueness related to the features of the biocatalyst (yield, substrate specificity, regioselectivity, and resistance to a particular reaction condition). The invention of glycosynthases is just one of the aspects that has thrust forward the research in this field. Protein engineering, metagenomics and reaction engineering have led to the discovery of an expanding number of novel enzymes and to the setting up of new bio-based processes for the preparation of glycosides. In this review, new examples from the last decade are compiled with attention both to cases in which naturally present, as well as genetically inserted, characteristics of the catalysts make them attractive for biocatalysis.

## 1. Introduction

In a brilliant, recently published analysis of the research-guided development in the field of biocatalysis during the last century, different authors recognized three historical waves of innovations that totally changed the field of biocatalysis to the present industrially accomplished level [1]. In a nutshell, while in the past limitations of an enzyme modeled the enzymatic process, today the enzyme can be engineered to be suitable for the process requirements. However this general bird’s-eye view cannot be specific for articulated contexts in which each single class of enzyme or selected enzymatic process is at the present state. In another similar general bird’s-eye analysis, Riva identified a long wave of successes still far from reaching the end in biocatalysis [2], due to the difficulties encountered in the shift from ‘classical’ processes to biobased ones. It is clear that exploiting natural catalysts to obtain selective transformations of non-natural substrates is far from being fully explored; among many others, the cases represented by the new concept of ‘third generation biorefineries’ [2] (producing chemicals from biomasses), or by the new glycoside hydrolases and other enzymes found in marine environments [3] have re-fostered new research trends in the field. As a matter of example, although investigation into hemicellulases as biorefining enzymes has been slow, as reported in a recent analysis [4], xylan-related biocatalysis has continued to make steady progress in many areas, including the discovery and characterization of a wide range of hemicellulases. Talking more specifically about biobased glycosynthesis, these studies are opening new prospects for the use of pentose sugars as building blocks for engineered pentosides as non-ionic surfactants or prebiotic food/feed ingredients.

Carbohydrates are involved in a broad range of functions in cell living systems. Structural roles and energy storage as functions were recognized during the first half of the last century while attainments in glycobiology and glycochemistry, during the last twenty years, have further revealed that carbohydrate parts of biomolecules (glycoproteins, glycolipids, *etc.*) are involved in important biological functions mainly related to cell recognition events [5,6,7]. It should not be neglected that carbohydrates are important molecules also in the technological domain. Synthetic carbohydrate-containing polymers have a wide range of applications in medical biotechnology [8]. A number of novel dietary carbohydrates produced by enzymatic syntheses have been introduced into food technology during the last decade [9]. In innovative fine chemical manufacturing solutions, straightforward synthesis of products is of interest (e.g., chromophoric oligosaccharides of strictly defined structure as valuable tools for the kinetic analysis of hydrolytic activities and for characterization of new exo- or endo-glycosidases). Finally, in cosmetics, prodrug action of enzymatically glycosylated natural lipophilic antioxidants is currently under consideration.

In general glycosylation is considered to be an important and quite special method for the structural modification of a compound. It allows the conversion of a lipophilic compound into a hydrophilic one changing pharmacokinetic properties or creating drug delivery systems. It could also be generalized that in a glycoside, the type of aglycon determines the application: long alkyl chains allow glycosides to possess useful properties as surfactants and emulsifiers; aglycon based on unsaturated alkyl chains are said to be valuable, as glycosides, for fungal infections or as antimicrobial agents; glycosides of peptides and steroids are used in antitumor formulations and cardiac-related drugs, respectively; and glycosides of flavors and fragrances are used as “controlled release” compounds [10,11].

Sugar units have more than one site through which the chains are extended. Each of these sites frequently shows very similar reactivity, thus the masking of reactive centers by protecting groups is essential in order to direct coupling through the right position. For this reason protection and deprotection steps of functional groups are in use extensively in the arsenal of the synthetic carbohydrate chemist; moreover ensuring the correct stereochemistry of the glycosidic linkage formed entails additional difficulty. Carbohydrate related synthetic chemistry can still be considered one of the well-explored branches of organic chemistry and very rich in significant and spectacular successes, although important alternative biomethodologies for assembling glycosidic linkages are presently known and acknowledged. It is worth noting that in comparison with chemical methods, enzymatic glycosylation is particularly useful for the modification of complex biologically active substances, when generally harsh conditions or use of toxic (heavy metals) catalysts are undesirable. Enzymes may represent an imperative choice in fields such as agriculture and food or cosmetics where chemical strategies are not acceptable [12]. In a very recent report detailing different examples of enzymatic glycosylation of small molecules, the authors concluded that challenging substrates require tailored catalysts, and the progress in the field of enzyme engineering and screening of new catalytic activities are both expected to result in new applications of biocatalytic glycosylations in various industrial sectors [13].

The enzymes responsible for the synthesis of glycosidic linkage have been recognized as transglycosylases and named glycosyltransferases, specifying the glycosyl donor and the reaction product. These enzymes transfer sugar moieties from activated donors to specific acceptors, forming stereochemically specific glycosidic bonds, and are responsible *in vivo* for the synthesis of most cell-surface glycoconjugates, using eight common sugar nucleotides as activated donors (Leloir pathway). Sugar phosphates act as donors for other glycosyltransferases (non-Leloir pathway). Another widespread group of enzymes, named glycoside hydrolases (glycosidases), exists; they are involved in the carbohydrate metabolism being responsible for the hydrolysis of glycosidic linkages; they can act as exo- or endo-glycosidases and are involved in a series of important biological events such as energy uptake, in processes inherent cell wall metabolism, in glycan processing during *in vivo* glycoprotein synthesis, *etc.* Based on historical grounds, glycoside hydrolases were implicated in most experimental observations during the early studies into the biological synthesis of glycosidic linkages at the beginning of the last century. Hence, the concepts of enzymatically promoted synthesis by both hydrolysis-reversal and glycosyl transfer soon appeared [14]. By the end of the 1980s, several research projects [15] testified the importance of different and interesting glycoside hydrolases, especially from the marine environment; their main application was centered on the structural identification efforts that faced the complexity of oligosaccharide structures before the instrumental exploit of 2D NMR and MS spectroscopy.

Different wild-type glycosidases and their modified versions are enzymes deserving new expectations in research and development today. Significant progress has been made in recent years for the application of these enzymes: even while the major breakthrough was the invention of glycosynthases, protein engineering, metagenomics and reaction engineering led to the discovery of an expanding number of novel enzymes and to the setting up of new bio-based processes for the preparation of important glycosides. This review will compile different examples where glycoside hydrolases are the key enzymes in the process.

## 2. Natural Enzymes for the Synthesis of Glycosidic Linkages

In chemical terms, considering both hydrolytic or synthetic aspects of esterases, glycosidases, phosphatases, transglycosidases and peptidases, the enzymatic mechanisms are based on displacement reactions and could be grouped together. This line of thought proved to be highly productive in historical terms, allowing the collection and rationalization of the amount of mechanistic data especially for glycosidases and transglycosylases. The stereochemistry of the mechanisms of glycoside hydrolases was analyzed by Koshland [16] more than 60 years ago and allowed the classification of inverting and retaining enzymes according to the anomeric configuration found in the product with respect to that in the starting substrate. Very recently, it has become clear that other mechanisms have evolved, such as the one based on elimination [17]. In 2010, in an interesting review on diversity of catalytic base nucleophile of glycoside hydrolases, it was reported that a variety of systems are used to replace this function, including substrate-assisted catalysis, a network of several residues, and the use of non-carboxylate residues or exogenous nucleophiles [18].

Glycosyltransferases-mediated reactions are thought to proceed via an oxocarbenium-ion-like transition state as proposed for glycosidase reactions on the basis of solid structural, mechanistic and *ab initio* molecular orbital calculations data [19]. Glycosyltransferases are catalysts for natural glycosylation reactions, known as “Leloir” glycosyl transferases (GT). Glycoside phosphorylases (GP), requiring glycosyl phosphates and transglycosidases (TG), employing non-activated carbohydrates (e.g., sucrose), are additional examples of synthetic enzymes. However glycoside hydrolases (GH) can also be used for synthetic purposes under either kinetic (transglycosylation) or thermodynamic (reverse hydrolysis) control. 

In this paragraph new examples related to the transfer of glycosyl residues between two oxygen nucleophiles are compiled with attention to those cases in which the natural characteristics of the catalyst make it attractive for biocatalysis (importance of molecular skeleton of substrates, yield, regioselectivity, resistance to particular reaction condition, *etc.*).

### 2.1. Interesting Transfer of Glycosyl Residues in Natural Enzymes for the Synthesis of Glycosidic Linkages

**Figure 1:**
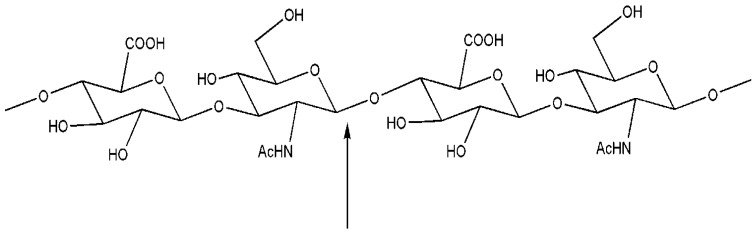
Tetrasaccharidic moiety of a hyaluronic acid chain and point of attach of hyaluronase for the hydrolysis and transglycosylation reactions.

Hyaluronic acid, synthesized by hyaluronan synthases, is a biopolymer abundant in extracellular matrices that is degraded by hyaluronidases. Biocompatibility and biodegradability of this polymer and of related small derivatives are of great interest in pharmaceutics in a number of molecular devices for drug delivery. Bovine testicular hyaluronidase (BTH) is a commercially available hyaluronidase preparation that has long been considered a prototype of mammalian hyaluronidases. Presumably all mammalian hyaluronidases can catalyze hydrolysis as well as transglycosylation reactions of hyaluronic acid fragments (Figure 1). In the case of BTH, the hydrolysis is favored at acidic pH values, while transglycosylation occurs preferentially at neutral pH and at low NaCl concentrations. The availability of recombinant expression systems for the production of purified human hyaluronidases PH-20 and Hyal-1 has facilitated the first detailed analysis of the enzymatic reaction products for these two enzymes. HA hexasaccharide, which is generally accepted as being the minimum substrate of BTH, is not a substrate of recombinant human PH-20 and Hyal-1 as recently demonstrated [20] although BTH and PH-20 belong to the same type of hyaluronidase. Interestingly, HA octasaccharide can be used as the substrate of Hyal-1 at pH 3.5. The substrate was converted quickly at concentration between 25 µM to 1 mM; above 1 mM weak substrate inhibition was observed. The study of transfer reactions, selectivities and yields are all features of interest for a possible use of these enzymes in biocatalytic steps for the manipulation of important biomolecules [21]. 

Additional new examples of this enzyme can be derived from other environments: the venoms of two classes of fish, freshwater stingray (members of the genus Potamotrygon) and stonefish (members of the genus Synanceia), contain, along with proteinaceous toxins, also hyaluronidases. These proteins are considered as spreading factors that facilitate the tissue diffusion of toxins by degrading hyaluronan; owing to this quick action it can possess very interesting features for biocatalysis [22].

**Figure 2:**
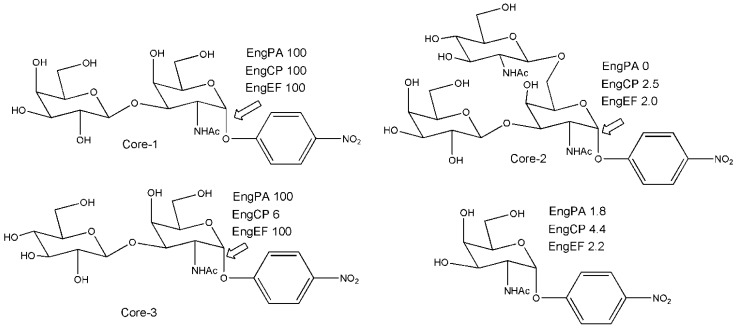
Substrates and percentages of products liberated at the link indicated by each single enzyme.

Sensitive and reliable enzymatic tools for the analysis of glycan chains are needed. The O-linked glycans are attached to serine or threonine through the GalNAc residue at the reducing end. The hydrolysis of the O-glycosidic α-linkage between GalNAc of the disaccharide Gal-β-1,3-GalNAc- and threonine or serine, is catalyzed by endo-α-N-acetylgalactosaminidase (EC 3.2.1.97). Most of the enzymes of this type are strictly specific for the disaccharidic structure and have no action on substrates with longer or different glycosyl chains. Using the protein sequence of a known endo-α-GalNAcase from *B. longum*, four potential sequences were found from BLAST (Basic Local Alignment Search Tool) search. Cloned and expressed proteins were purified and characterized [23]. Substrate specificity was investigated on aryl substrates as indicated in Figure 2 or by using natural glycoproteins. All three new enzymes are active on Core 1 substrate (Gal-β-1,3-GalNAc) while only two of them (EngEF and EngPA) were active after 24 h on Core 3 disaccharide (Glc-β-1,3-GalNAc). Interestingly, these enzymes acted also as transglycosylating agents; when reacted with simple alkanols (from methanol to nonanol) as acceptors, Core 1 and Core 3 acted as donor disaccharides in test reactions. Although the yields as judged by TLC analysis were low (at 0.8-1.6 mM donor and 13% v/v alkanols), positive reactions were observed up to 4–5 alkanol carbon atoms. As stated by the authors in the concluding remarks, the action of EngEF and EngPA enzymes acting on Core 3 in addition to Core 1 O-glycans could make these enzymes powerful tools for the release of O-glycan sugars from glycoproteins. More importantly they can also be used as templates in future protein engineering experiments for a possible creation of endo-α-GalNAcases capable of acting on O-linked glycans, regardless of their sugar composition.

**Figure 3:**
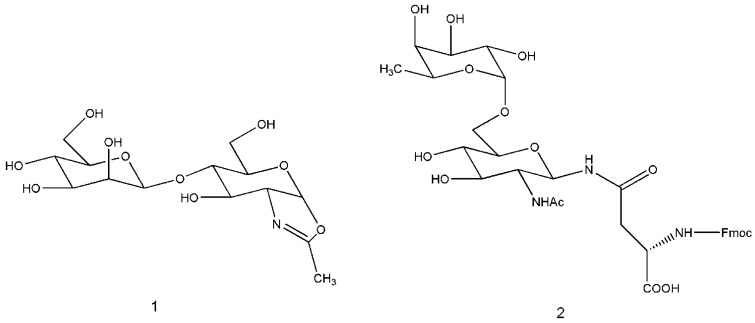
Typical oxazoline substrate 1 reacting with Endo-M or Endo-A and capped α-1,6-fucose (1,6-fucosyl-GlcNAc derivative) used as acceptors for transglycosylation by Endo-F2 and Endo-F3 [25].

One of the last outstanding results in transglycosylation reactions in the last decade is, without any doubt, the recognition that endo-β-N-acetylglucosaminidase can transglycosylate a large oligosaccharide onto various glycosyl acceptors. In 2001, the Shoda group reported on the synthesis of a novel disaccharide possessing a 1,2-oxazoline moiety and tested it with a series of enzymes for transglycosylation activity. Typical oxazoline substrate 1, as depicted in Figure 3, reacted with Endo-M or Endo-A from *Mucor hiemalis* or *A. protophormiae*, respectively and the acceptor GlcNAc-β-1-O-pNP for the synthesis of a trisaccharidic derivative in high yield [24]. These interesting biocatalysts (Endo-M or Endo-A), which belong to GH family 85 were inactive when they have to use acceptors capped with an α-1,6-fucose (1,6-fucosyl-GlcNAc derivative as acceptors for transglycosylation). But more recently, the Huang group, screening various endo-β-N-acetylglucosaminidases using appropriate synthetic oxazoline donors and compound 2, Figure 3, as acceptor substrate, found that endo-β-N-acetylglucosaminidases from *Flavobacterium meningosepticum* (including Endo-F2 and Endo-F3), were able to glycosylate α-1,6-fucosylated GlcNAc derivative to provide natural, core-fucosylated complex type N-glycopeptides [25]. The product(s) were isolated in high yield by HPLC and this efficiency of transglycosylation is quite impressive, given the fact that only two-fold of the donor substrate was used.

Starting from the known consideration that extracellular glycosidases from fungi, (used to access low molecular weight sugars by their hydrolytic action on polymeric substrates), must possess high flexibility in substrate specificity, the group of Kren recently investigated new enzymes in which the 4-hydroxy moiety of the pyranose ring of the substrate is not essential for binding to the enzyme active site. In their report they emphasize also the transglycosylation activity of β-N-acetylhexosaminidase from *Talaromyces flavus*. The results reported on the production of three novel 4′-deoxy-disaccharides prepared in high yields (52% total disaccharide fraction) starting with phenyl 2-acetamido-2,4-dideoxy-substrate. The conditions of the transglycosylation reaction were first optimized on an analytical scale by varying the concentrations and ratios of the reaction components and identifiying the following reaction conditions as the most efficient: 75 mM donor, 300 mM acceptor, incubation for 5–6 h at 35 °C [26]. 

The enzymatic formation of β-D-fucosides is hardly described in the scientific literature. An extraordinarily broad substrate specificity for both hydrolysis and transglycosylation was exhibited by a glycosidase isolated from the China white jade snail. Acceptor specificity for monosaccharides and transfer efficiency have both been investigated for this promising enzyme [27] and from the results obtained, the authors indicated a very high transfucosylation efficiency using p-nitrophenyl derivative of β-D-fucose at 10 mM and acceptors (at 20–100 mM) such as glucose (88% yield) and xylose (93% yield); the interglycosidic linkage formed with glucose is β-1,6 thus they proposed this biocatalyst as a useful candidate for disaccharide synthesis. The Fuc-β-Xyl disaccharide formed in other reactions is a building block for the synthesis of asterosaponins of marine origin, although in natural compound the interglycosidic linkage is different (Fuc-β-(1-2)-Xyl) [28].

In screening a suitable biocatalyst for galactosylation of nucleosides, Ye *et al.* very recently found that β-galactosidases from *Kluyveromyces lactis* (Sigma, USA) and a crude glycosidase extract of apple seeds, had high hydrolytic activities toward oNPGal, but could not catalyze transglycosylation reactions between oNPGal and 2β-deoxynucleosides [29]. Indeed, only very few reports have appeared in the literature in the last 10 years leading with successful glycosidase-mediated nucleoside glycosylation: (i) after low-yielding results using β-galactosidase from *Aspergillus oryzae* [30], the first successful approach, which appeared in 2007, was (ii) the convenient synthesis of β-galactosyl derivatives of antiviral and anticancer nucleosides, utilizing the purified β-galactosidase activity from the hepatopancreas of *Aplysia fasciata* [31] and the second valid method is (iii) the regioselective galactosylation of floxuridine (FUdR) catalyzed by a commercial β-galactosidase from bovine liver with a high yield (75%) and an excellent 5-β-regioselectivity (>99%) [32] using o-nitrophenyl β-D-galactoside as glycosyl donor. Such desirable products were synthesized with satisfactory yields (41–68%) and moderate to high 5β-regioselectivities (87–100%), also by using the crude enzyme extract.

In the hepatopancreas and visceral mass of the mollusc *Aplysia fasciata* a wide range of glycoside hydrolases (α-glucosidase, β-galactosidase, β-glucosidase, β-mannosidase and others) were found and successfully used for the hydrolysis and synthesis of glycosidic bonds. The formation of α-D-oligoglucosides from maltose (up to tetra- and pentasaccharides) was studied in detail [33] and it was also demonstrated that the enzyme was able to form mono- and di- and poly-glucosides of different externally added acceptors [3 and references cited therein]. This activity has been applied in the very recent chemoenzymatic synthesis of α-6-sulfoquinovosyl-1,2-O-diacylglycerols, a class of natural lipids that have attracted biomedical attention as possible antitumors, antivirals, and immunomodulators. This synthesis started with the enzymatically controlled transfer of glucose from maltose (10:1 molar ratio) to (*rac*)-1,2-O-isopropylidene glycerol. The 1,2 glycerol acetonide conversion of 59% in 29 h was obtained and reaction products were exclusively 3-α-glycosyl derivatives of 1,2-O-isopropylidene glycerol. In particular 3-α-glucosyl-1,2-O-isopropylidene glycerol was produced as the major component (30% yield) together with a mixture of di- and tri-saccharide analogues (23 and 6% yields, respectively). Under similar conditions the transglycosidase of *Aspergillus niger* and the α-glucosidase of *Bacillus stearothermophilus* gave 3-α-glucosyl-1,2-O-isopropylidene glycerol with yields below 2%. This comparison suggests that marine enzymes may offer a viable option for the synthesis of different types of glycolipids [34].

There are other glycoside hydrolase activities that are hardly used in synthesis. A thermostable α-L-arabinofuranosidase was reported in 2002 for its ability to perform transarabinosylation reactions for the synthesis of different α-L-arabinofuranosides of various alcohols. The same enzyme was later reported as a useful biocatalyst for the synthesis of p-nitrophenyl α-L-arabinofuranosyl-(1-2)-α-L-arabinofuranoside, p-nitrophenyl β-D-xylopyranosyl-(1-2)-β-D-xylopyranoside, p-nitrophenyl β-D-xylopyranosyl-(1-3)-β-D-xylopyranoside and benzyl α-L-arabinofuranosyl-(1-2)- α-D-xylopyranoside. Such a disaccharidic motif could be used as the reference compound for the analysis of hemicellulase action and for raising antibodies to well-defined motifs for immunochemical-based analyses of plant cell walls [35]. The same group reported also the synthesis of galactofuranosides using the above catalyst [36]. β-D-galactofuranose is a cell wall constituent in several pathogenic species including *Mycobacterium tuberculosis* and *leprae*, agents of tuberculosis and leprosy, respectively. The synthesis of galactofuranose analogues acting as inhibitors of the biosynthetic enzymes (UDP-galactopyranose mutase and galactofuranosyl transferases) or the synthesis of galactofuranosides that could be used for the elaboration of vaccines, is important for this aspect. Authors reported also on the chemical synthesis of p-nitrophenyl galactofuranoside used as donor and the low yield obtained (16%) could act as an additional center of interest for the synthetic aspect of this kind of enzyme.

An interesting case of the effect of solvents in transglycosylation reactions has been studied recently for the synthesis of N-acetyl-D-lactosamine, Gal-β-1,4-GlcNAc (LacNAc), catalyzed by the β-galactosidase from *Thermus thermophilus* (TTP0042). The authors studied the percentages of all products formed (self-condensation products, hydrolytic originating galactose and transfer regioisomers). The low yield (about 20%) of LAcNAc when the reaction is performed in buffer is increased by up to 91% when the process takes place in the presence of glycerol based solvents. According to conformational studies of the enzyme structure by circular dichroism and fluorescence, the authors concluded that in the presence of these solvents the enzyme modifies secondary and tertiary structure and this may also be the cause of some additional regioselectivity changes observed [37]. Unfortunately no molecular details were furnished, indeed no clear relationship between the very different solvent structures used and the regioselectivity change was envisaged. 

**Figure 4:**
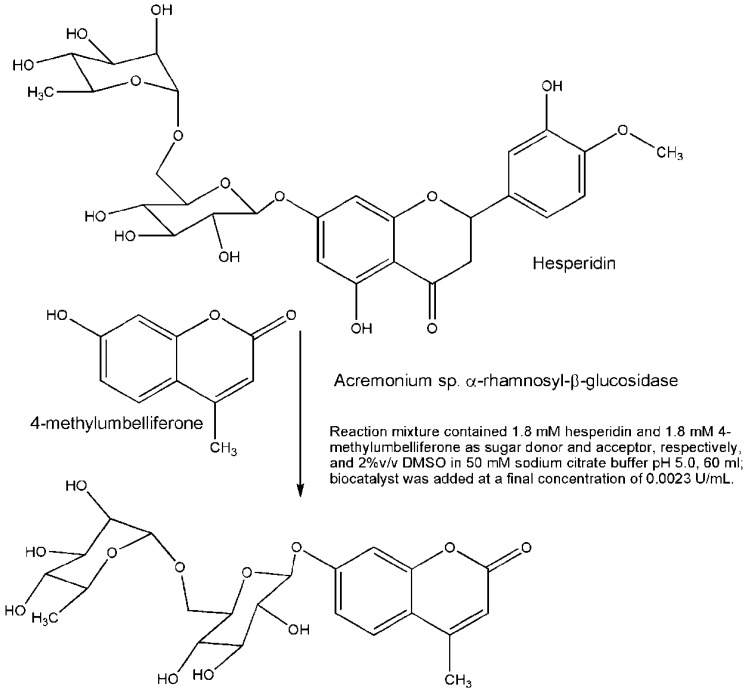
Transrutinosylation performed by diglycosidase isolated from Acremonium sp.

A new and interesting fungal diglycosidase was isolated from *Acremonium* sp. It has an α-rhamnosyl-β-glucosidase activity with transglycosylation potential of the entire disaccharide (rutinose) moiety from natural products to different acceptors. This enzyme allowed the synthesis of the diglycoconjugated fluorogenic substrate 4-methylumbelliferyl-rutinoside. The synthesis was performed in one step from the corresponding aglycone, 4-methylumbelliferone, and hesperidin as rutinose donor with a 16% yield regarding the sugar acceptor [38]. Despite the low yield, the fluorogenic substrate formed, 4-methylumbelliferyl rutinoside (Figure 4), which is not commercially available, is important for the study of diglycosidases since it allows the detection of enzymes specific for rutinose mobilization. Although the activity of this enzyme could seem quite specific for a broad interest, we must bear in mind its potential use for industrial processing of plant-based foods and the ability to transglycosylate rutinosyl units from abundant and inexpensive by-products of citrus industry.

## 3. Engineered Enzymes

From 2004 to 2013 PubMed indexed 78 hits having the words glycosynthase(s) in the title, including review articles that accounted for ca. 20%. Similar figures can be found in Scopus and in Web of Knowledge. Using data published up to the mid-term of 2003, Perugino *et al.* compiled a review [39] accounting for 11 different glycoside hydrolases from bacteria, eukarya and archaea, that were modified as efficient glycosynthases. In 2012, in an update report from the same lab [40] a quadrupled value was found. Although these lists were declared not exhaustive, a similar number is also reported in the review of 2011 from Wong labs [41]. Indeed a number of excellent reviews periodically survey the most recent achievements in the field of glycosynthases from different perspectives, such as their production and application to the synthesis of glycoconjugates. The obvious success is due to quantitative yields that can be reached in reactions using these active-site modified glycoside hydrolases. The glycosynthase approach is certainly of great help while keeping molecular diversity offered by different natural glycoside hydrolases, including features such as resistance to temperature, organic solvent, *etc.,* making this aspect very interesting for the exploitation of these enzymes in biocatalysis. A survey of all these examples will not be repeated here, where instead just one case of a spectacular glycosphingolipids synthesis is reported below. 

The focus in the remaining part of this paragraph, dedicated to engineered enzymes, is on those cases where genetic modifications could help in different manner than increasing yield by glycosynthase philosophy.

### 3.1. Preparative Glycosphingolipids Synthesis Operated by Glycosynthase

**Figure 5:**
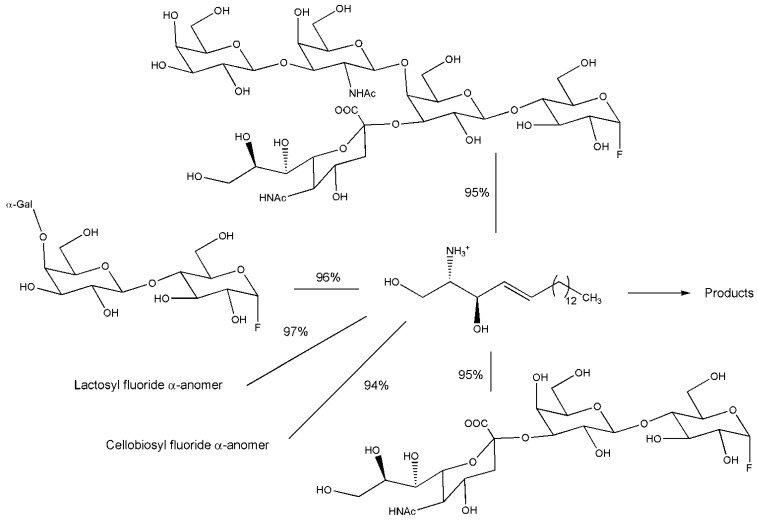
Reaction yields for EGCII glycosynthase with various glycosyl fluoride.

Pharmacological interest for glycosphingolipids, a class of glycolipids based on the aminodiol sphingosine, is high since these compounds have numerous biological functions in processes of human physiology [42]. Gangliosides are a subclass of glycosphingolipids containing also sialic acid in the oligosaccharide moiety, composed of different sugars. Certain gangliosides are involved in viral infections mechanisms. Commercial sources for glycosphingolipids rely on isolation of compounds from natural sources, such as bovine brain and canine blood. This is impractical for large-scale preparation, as well as posing risks for contamination and requiring great effort for extensive purification. A spectacular example of application of glycosynthase technology for high yield production of different gangliosides has been recently reported [43]. The work is based on the transformation of a natural hydrolytic enzyme, the endoglycoceramidase II (EGC II) from *Rhodococcus* strain M-777, in a glycosynthase. This glycosynthase version of the enzyme with Ala, Ser or Gly as substituents of active site nucleophile, is not capable of performing hydrolysis, but it can react in synthetic mode using α-anomer of different oligosaccharidic fluorides (di- to pentasaccharides) in the presence of acceptors such as D-erythro-sphingosine or sphingosine analogues (Figure 5). Yields from 70 to 100% were obtained for the lyso product on 300 mg scale.

Reactants so structurally different could pose problems, but the solubility of the hydrochloride salt of the sphingosine in aqueous buffer (25 mM after sonication at 37 °C) is considered by the authors a benefit for possible molecular diversity that can be obtained with successive acylation steps with structurally diverse acyl chains. In fact, N-acyl substituents in ceramides affects biological activity of these compounds to some extent, and the acyl substituent has also been identified as a convenient position for conjugation of non-lipid substituents, including fluorescent tags [44].

### 3.2. Engineering Glycoside Hydrolases not at the Active Site

Engineering the active-site environs is a technique that has been used to enhance the transglycosylation activity of glycosidases and to modify other features of interest in biocatalysis. When structural architecture of the active site is not known, a site-specific “randomization” approach could be used in which an amino acid residue near the catalytic site can be replaced by each of all other amino acids to screen mutant enzyme(s) of interest. This strategy was used to obtain α-glucosidase variant(s) for increasing the production of theanderose, a trisaccharide obtained from sucrose by α-glucosylation to the C6 of glucose. An amino acid residue (Gly273 or Thr272) near the putative catalytic site (Glu271) of this *Bacillus* α-glucosidase was replaced by all other naturally-occurring amino acids thus increasing the transglucosylation activity. The highest specificity for theanderose formation (*i.e.*, the highest content of theanderose in the reaction product) was obtained with the isoleucine containing mutant (T272I), which showed 1.74 times higher productivity (per sucrose-hydrolyzing unit) of theanderose than that of the wild-type enzyme. The authors concluded, however, that elucidation of three-dimensional structures should help to understand the details of the mechanism eliciting the specificity obtained [45]. More recently, the transglycosylation/hydrolysis ratio was shown to be 3- and 8-fold increased with two mutants of a different enzyme, the *Thermotoga neapolitana* β-glucosidase. The asparagine mutant (N291T) of this enzyme showed also altered regioselectivity. In particular TLC analysis of the transglycosylation products indicated that while the mutant retained its β-1,3 regioselectivity, β-1,4 and β-1,6 selectivities were lost when pNPG and arbutin were used as a donor and acceptor, respectively [46]. In another case, Feng *et al.* in 2005 described directed evolution applied to the β-glycosidase of *Thermus thermophilus* to increase the ability of this enzyme in transglycosylation reaction. The most efficient mutations of phenylalanine and asparagine (F401S and N282T), were located just in front of the acceptor subsite and the authors suggested that repositioning of the glycone in its subsite together with a better fit of the acceptor in the acceptor subsite, might favor the attack of a glycosyl acceptor in the mutant at the expense of water; this conclusion was based on molecular modeling techniques. They also concluded that their results suggest that directed evolution of the glycosidases in transglycosidases could be an alternative to the glycosynthase strategy; in fact, for certain mutants, synthesis by self-condensation of nitrophenyl glycosides became nearly quantitative [47]. However when the asparagine (N282T) mutant was analyzed with external added acceptor pNPGlcNAc using oNPGal as donor, the NMR study of kinetic formation of transglycosylation products showed that those due to self-condensation (Galβ1-3Gal-oNP and Galβ1-6Gal-oNP) are kinetically favored over the condensation product Galβ1-4GlcNAc-pNP, suggesting that oNPGal is a better acceptor than pNPGlcNAc; competition between self-condensation and condensation could be responsible for the moderate yield of the desired product Galβ1-4GlcNAc-pNP. Based on the analysis of catalytic context, the authors studied *in silico* the complex alanine mutant-acceptor (A221W)/pNPGlcNAc establishing that the docking of the acceptor is not perturbed by the mutation, compared to the WT case. Donor docking was also studied with A221W mutant complexed with oNPGal to analyze precisely the perturbation as compared with WT. In WT enzyme there is enough room for oNPGal in the acceptor sites allowing the self-condensation reaction. In the A221W mutant, a drastic steric conflict occurs between the galactose ring and the tryptophan substituting alanine and this effect was seen by the authors as the most promising for preventing the self-condensation reaction. The mutation A221W was thus introduced by directed mutagenesis. The analysis of products and relative yields of this double mutant N282T/A221W shows that these results are consistent with the MM calculations. The authors proposed their approach as convenient in that relatively stable activated sugars can be used [48]. While this work shows the value of a rational approach to eliminate the side effects of transglycosylation reactions, a thermophilic glycosynthase from *Sulfolobus solfataricus* was indeed shown to act in presence of external formate on stable activated sugars such as oNP-glyco donors [49].

A random mutagenic approach coupled to a screening procedure was applied in another thermophilic case in nature. The screening was based on the reduction of the hydrolysis of a potential transglycosylation product (lactosucrose, β-D-galactopyranosyl-(1-4)-D-sucrose) formed in presence of sucrose, thus providing mutant enzymes possessing improved synthetic properties for the transglycosylation [50]. The application of thermostable β-galactosidases such as the β-galactosidase BgaB from *Geobacillus stearothermophilus* is of interest for transgalactosylation because at higher temperatures higher lactose concentration can be used to favor the synthesis.

A complete change of product profile of the reaction was observed for one of the mutants obtained by site directed mutagenesis of α-amylase of *Bacillus amyloliquefaciens*. The Val289 residue substituted with tyrosine showed less than 15% activity compared to the wild-type, but it acquired transglycosylation activity, producing longer oligosaccharides. The analysis of all mutants produced led to the conclusion that changes in the hydrolytic property of α-amylase may be due to factors like the geometry and electrostatics of the environment around the active site [51].

It could be of interest in this paragraph to mention the development of methodologies for the screening of large libraries of mutant enzymes. In a paper of 2009 Konè *et al.* reported on digital screening methodology as a system dedicated to the screening for sugar-transfer activity [52], which gave great impetus to the study of glycosidases with efficient transglycosidases activity as an alternative to glycosyltransferases or glycosynthases. Recently, from genomic and metagenomic program results, protein sequences with unknown functions are increasing, thus effective screening methodologies have become an important aspect of this research. In the field of polysaccharide degrading enzymes, a profiling method reported in 2012 by the Helbert group is worth noting. Polysaccharidases are important enzymes that are used as specific tools to improve conversion of lignocellulosic biomass into sugar monomers prior to ethanol production, for: degradation of microbial polysaccharides, obtaining bioactive materials, for structural determination of unknown complex polysaccharides structures, *etc.* The profiling strategy is based on a series of filtrations necessary to eliminate any reducing sugars not directly generated by enzyme degradation. After enzymatic action, filtrates are assayed with a ferricyanide solution to reveal the reducing sugars produced by glycoside hydrolases or polysaccharide lyases; however matrix-assisted laser desorption ionization mass spectrometry (MALDI-MS), presenting several unique advantages for the structural characterization of degradation products of carbohydrates, is also considered as providing an effective methodology [53,54].

## 4. Conclusions

Although not exhaustively catalogued in this review, the scientific literature of the last decade, with regard to the process of enzymatic preparation of glycosides, enables the reader to conclude that research for uniqueness, in terms of enzymatic features, is still active. This is in contrast with the tempting generalization about enzymes as plastic biomolecules fully receptive to be engineered by appropriate changes, although this plasticity has been successfully demonstrated in some examples [1]. The list of naturally “unique” enzymes cited in this review includes examples found among the most promising subjects of biocatalytic applications: (i) hyaluronidases for producing and/or transferring hyaluronic oligosaccharides of pharmaceutical interest or (ii) disaccharidases such as endo-α-N-acetylgalactosaminidase (for the analysis of glycan chains) and (iii) the fungal rutinosidase used in the synthesis of related fluorogenic derivative of 4-methylumbelliferone, are of specific interest. Among engineered enzymes, even though glycosynthases have to be seen as the most successful approach to reach quantitative transfer yields in transglycosylations, engineered enzymes examples with mutations only in the active-site environ (and not at active site aminoacids), demonstrated that biocatalytic features are well manipulated also in this way, thus enlarging competence to regioselectivity and or specificity for heteroacceptors and not only to reaction yield as devised for glycosynthases.

This overview should increase the interest from biocatalyst practitioners towards both naturally unique enzymes and for infrequent mutations capable of inducing biocatalytically interesting features.
